# Interstitial ectopic pregnancy: A rare challenged case report

**DOI:** 10.1016/j.ijscr.2025.110938

**Published:** 2025-01-23

**Authors:** Youssef Essebbagh, Khadija Errmili, Maha Lhaloui, Soukaina Mouimen, Najia Zeraidi, Aziz Baidada

**Affiliations:** Gynaecology-Obstetrics and Endoscopy Department, Maternity Souissi, University Hospital Center IBN SINA, University Mohammed V, Rabat, Morocco

**Keywords:** Interstitial pregnancy, Case report, Rare phenomenon

## Abstract

**Introduction:**

Interstitial pregnancy, a rare form of ectopic pregnancy, occurs when implantation happens in the uterine wall's intramural tubal segment. Though uncommon, it carries high risks like hemorrhagic rupture, necessitating early diagnosis and prompt management.

**Case presentation:**

A 33-year-old primigravida patient with no medical or surgical history and no particular risk factors presented with acute abdominal pain and minimal metrorrhagia. Physical examination revealed minimal uterine bleeding and slight pain on uterine mobilization. Ultrasound showed an enlarged uterus with a visible cavity and the presence of a right lateral uterine mass in favor an interstitial pregnancy. Laparotomy, which confirmed the diagnosis was done with a salpingectomy and cornuotomie.

**Discussion:**

Interstitial pregnancy is a rare and potentially life-threatening form of ectopic pregnancy, constituting only 2.4 % of cases. It differs anatomically from other types like angular or cornual pregnancies and poses a high risk of uterine rupture and severe hemorrhage due to delayed diagnosis. While β-hCG kinetics may offer clues, diagnosis primarily relies on transvaginal ultrasound, with MRI or 3D ultrasound as supplementary tools in complex cases. Treatment can be medical or surgical with laparoscopy preferred when available. Obstetric outcomes are generally favorable, with some cases supporting elective cesarean delivery as a precaution.

**Conclusion:**

Early diagnosis is crucial to prevent the potentially life-threatening progression of interstitial pregnancy; therefore, this condition should be considered in women who present with abdominal pain and/or vaginal bleeding during the first trimester.

## Highlights

• Interstitial ectopic pregnancy is the most dangerous form of tubal pregnancy.
• Diagnosis is a challenge due to non-classic presentation.
• Asymptomatic until catastrophic
• Diagnosis on routine ultrasound antenatal screening
• Main treatments are medical with methotrexate or surgical.

## 1 Introduction

Interstitial pregnancy is a rare form of ectopic pregnancy that occurs when the fertilized egg implants in the intramural segment of the fallopian tube (a canal approximately 0.7 mm wide and 1–2 cm long), often within an anatomically normal uterus [1]. Although less common than tubal pregnancies, it accounts for approximately 2.4 % of ectopic pregnancies and is associated with a high risk of complications, including hemorrhagic rupture. Early diagnosis and appropriate management are crucial to reducing the morbidity and mortality associated with this condition [2].

In this work, we present a case of interstitial pregnancy, through this case and in light of a review of the literature, we emphasize the key features of this condition, particularly the diagnostic methods, therapeutic management, and prognosis of this rare complication.

Our work has been reported in line with the SCARE Guidelines 2023 criteria [3].

## 2 Case report

We report the case of a 33-year-old primigravida patient with no medical or surgical history and no particular risk factors. She presented to the gynecological emergency department with acute abdominal pain lasting for 12 h, in a context of 7 weeks of amenorrhea and minimal metrorrhagia.

Upon examination, the patient had a blood pressure of 120/70 mmHg with tachycardia at 100 bpm. Her abdomen was soft on palpation, with no guarding or tenderness. A speculum examination revealed minimal uterine bleeding, while a bimanual pelvic examination identified slight pain on uterine mobilization without detecting a lateral uterine mass.

**Fig. 1:**
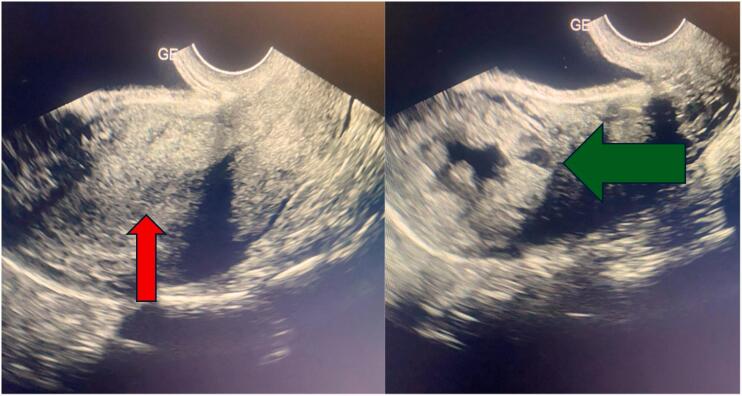
Red arrow uterine vacuity line & green arrow latero uterine mass.

A transvaginal pelvic ultrasound showed an enlarged uterus with a visible cavity line, thickened endometrium, and the presence of a right lateral uterine mass. The mass distorted the external contours of the uterus, appeared heterogeneous with an anechoic center and an echogenic rim, and was entirely surrounded by a myometrial layer, measuring 2.9 × 2 cm in favor an interstitial pregnancy, both adnexa were normal, and there was no pelvic effusion. Fig. 1.

Biological β-hCG levels were 3500 IU/mL, and the remainder of her laboratory results were normal.

**Fig. 2:**
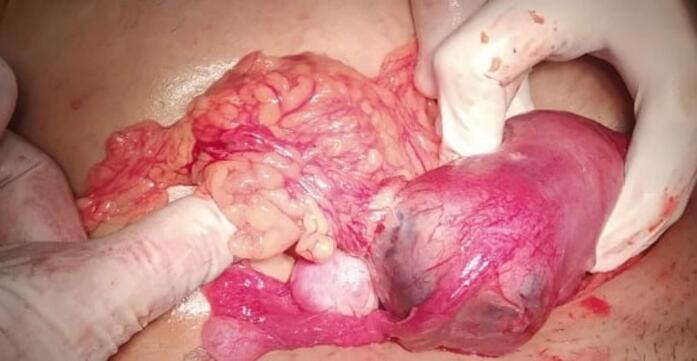
Intraoperative image showing interstitial pregnancy with omental adhesion.

**Fig. 3:**
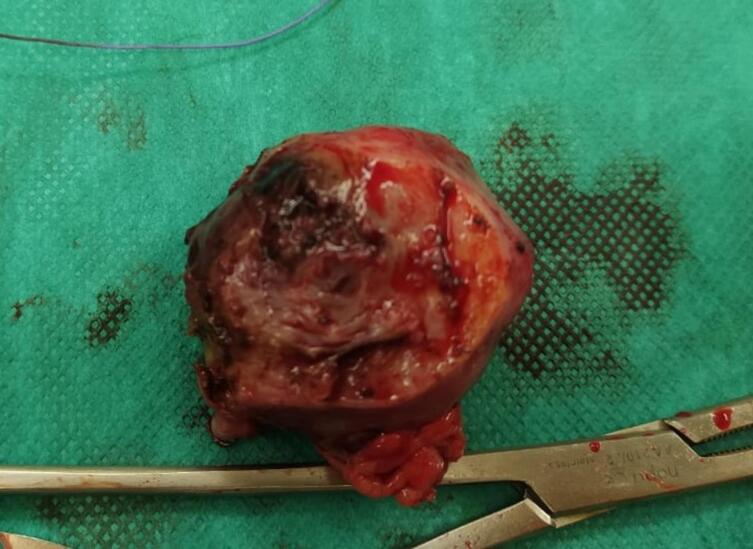
Corouotomy surgical piece.

The patient underwent laparotomy, which confirmed the diagnosis. Surgical management included a salpingectomy with cornuotomy and closure of the myometrium using Vicryl 01 sutures, estimated blood loss was approximately 300 mL. The contralateral fallopian tube was healthy, and there was no adhesion Fig. 2, Fig. 3.

Postoperative recovery was uneventful, and the patient was discharged on postoperative day 3.

She was followed at our institution for two years and subsequently conceived spontaneously. She had an uneventful intrauterine pregnancy carried to term. As a precaution, an elective cesarean section was performed at 38 weeks of gestation, during which no uterine dehiscence was reported.

## 3 Discussion

Interstitial pregnancy is a rare form of ectopic pregnancy, which encompasses various entities including: ovarian, abdominal, cornual, angular, and interstitial pregnancies, the last three are often grouped under the term “cornual pregnancies,” yet they differ anatomically: angular pregnancies develop intrauterine, sparing the tube and tubal ostium; interstitial pregnancies involve implantation in the tubal portion embedded within the myometrial thickness of the uterus; and cornual pregnancies occur in a rudimentary uterine horn in cases of a bicornuate uterus or on a tubal stump after salpingectomy [4].

Tubal ectopic pregnancies account for 93 % of ectopic pregnancies, while ovarian or abdominal pregnancies represent 4.5 %, and interstitial pregnancies (IP) make up only 2.4 % [1,5].

The primary risk factors for ectopic pregnancy include chronic pelvic infections (often due to sexually transmitted infections), which may lead to adhesions and anatomical anomalies, previous ectopic pregnancy or tubal surgery, smoking, and intrauterine device use. However, in our case, none of these risk factors were identified [6,7].

The clinical presentation of interstitial pregnancies is often subtle, with symptoms such as pelvic pain typically appearing later, contributing to delayed diagnosis. This delay increases the risk of sudden uterine cornual rupture and severe hemorrhage, often manifesting with catastrophic clinical signs in the context of hypovolemic shock. In our patient, acute-onset pelvic pain was the only clinical symptom [7,8].

Diagnosis is sometimes guided by atypical β-hCG kinetics but primarily relies on transvaginal pelvic ultrasound. This modality can identify an abnormally lateralized gestational sac surrounded by myometrium and protruding from the lateral border of the uterine fundus. Timor-Tritsch outlined three essential ultrasound criteria [9]:

• An empty uterine cavity;
• A gestational sac located more than 1 cm away from the uterine cavity;
• A thin myometrial layer surrounding the sac.

All three criteria were observed in our patient.

The role of 3D ultrasound is not well established in the literature but could provide more precise visualization of the trophoblastic crown (using 3D power Doppler) and help differentiate between a tubal and interstitial ectopic pregnancy. MRI serves as a more precise alternative for confirming the diagnosis and topographical localization of rare forms of ectopic pregnancy. It is especially helpful in complex cases with ambiguous ultrasound findings and can differentiate reproductive organs from potential lesions in other pelvic structures. MRI also plays a key role in suspected cases of accreta [[9], [10], [11]].

The treatment of interstitial pregnancies can be medical or surgical, depending on the clinical presentation and the patient's general condition. Methotrexate is frequently used as a medical treatment in non-ruptured cases, as it inhibits cell proliferation and allows for the resolution of trophoblastic tissue without invasive surgical intervention. However, close monitoring is crucial to ensure an appropriate decline in β-hCG levels [[12], [13], [14]].

In cases of rupture or hemodynamic instability, immediate surgical intervention is necessary, typically involving salpingectomy with cornuotomy. While laparoscopy is generally preferred due to its minimally invasive nature, the choice of approach depends on the surgical team's expertise and available resources [12,13,15]. In our case, the patient opted for surgery after declining medical treatment with methotrexate. Due to the unavailability of laparoscopy, a laparotomy was performed.

Regarding subsequent obstetric outcomes and mode of delivery, there is no consensus or formal recommendations in the literature. Many authors advocate for elective cesarean delivery as a precaution, although some reports describe successful vaginal deliveries without complications [12,14].

Our patient was followed up during a subsequent spontaneous pregnancy carried to term without complications. An elective cesarean section was performed at 38 weeks of gestation, with no evidence of uterine dehiscence.

## 4 Conclusion

Interstitial pregnancy is a rare but potentially life-threatening condition that requires meticulous attention during diagnosis and treatment. A multidisciplinary approach, including thorough ultrasound monitoring and appropriate clinical evaluation, is essential to optimize clinical outcomes. Raising awareness of this condition among healthcare professionals can help reduce severe complications and improve maternal outcomes.

## Consent

Written informed consent was obtained from the patient to publish this case report and accompanying images. On request, a copy of the written consent is available for review by the Editor-in-Chief of this journal.

## Ethical approval

This case report is exempt from ethical approval in our institute, Ibn Sina University Hospital Center.

## Guarantor

Youssef Essebbagh

## Research registration number

Not applicable.

## Funding

The authors declare that there is no funding or grant support.

## Author contribution

Youssef Essebbagh, Soukaina Mouimen: performed surgery, paper writing and editing.

Najia Zeraidi, Aziz BAIDADA: literature review, Supervision.

Youssef Essebbagh, Khadija Errmili, Maha Lahloui: Manuscript editing, picture editing

## Conflict of interest statement

The authors declare that they have no competing interests relevant to the content of this article.
